# Predicting 90-day mortality in patients with HBV-ACLF: A comparison of machine learning models and logistic regression

**DOI:** 10.1097/MD.0000000000050789

**Published:** 2026-09-25

**Authors:** Juan Liu, Wentao Zhu, Ting Deng, Yin Zhu, Qinglang Xu, Pei Shi, An Liang, Yuna Wang, Jiwei Fu, Xiaoping Wu

**Affiliations:** a Jiangxi Medical Center for Critical Public Health Events, The First Affiliated Hospital of Nanchang University, Nanchang, Jiangxi, China; b Department of Infectious Diseases, The First Affiliated Hospital of Nanchang University, Nanchang, Jiangxi, China; c Department of Cardiovascular Medicine, Jiangxi Provincial People’s Hospital, Nanchang, Jiangxi, China.

**Keywords:** 90-day mortality, acute-on-chronic liver failure, machine learning, prognosis

## Abstract

Acute-on-chronic liver failure (ACLF) is characterized by a high short-term mortality rate. Hepatitis B virus (HBV) infection is a major cause of ACLF in the Asia-Pacific region. This study aimed to compare the performance of multiple prediction models and identify a stable approach for predicting 90-day mortality in patients with HBV-associated ACLF (HBV-ACLF). Clinical data from 577 patients with HBV-ACLF admitted to 2 hospitals were retrospectively collected. Among them, 513 patients from 1 hospital were randomly divided into a training cohort and a testing cohort at a ratio of 6:4, while 64 patients from another hospital were included as an external validation cohort. Six prediction approaches were evaluated, including logistic regression (LR), least absolute shrinkage and selection operator regression (LASSO), support vector machine (SVM), decision tree (DT), random forest (RF), and K-nearest neighbor (KNN). Model performance was assessed using accuracy, the area under the receiver operating characteristic curve (AUC), sensitivity, specificity, positive predictive value, and negative predictive value. The RF model achieved excellent discrimination in the training cohort, with an AUC of 1.000. However, its performance declined in the testing cohort and external validation cohort, with AUC values of 0.912 and 0.728, respectively. In contrast, the LR model demonstrated more consistent predictive performance across the training, testing, and external validation cohorts, with AUC values of 0.905, 0.928, and 0.849, respectively. Overall, the LR model showed more consistent point estimates of discrimination across the internal testing and external validation cohorts than the more complex models. Although several machine learning approaches demonstrated good predictive performance, the LR showed the most stable and consistent performance across the testing and external validation cohorts. These findings suggest that increased model complexity does not necessarily translate into superior generalizability and that simpler, more interpretable models may provide robust prognostic prediction for patients with HBV-ACLF.

## 1. Introduction

Acute-on-chronic liver failure (ACLF) is characterized by deterioration of liver function due to an acute precipitating event or unrecognizable trigger in the context of underlying chronic liver disease. It is a short-term clinical syndrome of acute or subacute hepatic failure, with key manifestations including coagulation disorders, jaundice, ascites, and hepatic encephalopathy (HE).^[1-4]^ Chronic hepatitis B virus (HBV) infection is the primary cause of ACLF in the Asia-Pacific region, including China, exceeding more than 60% of total cases.^[5]^ Current effective treatments for ACLF include managing precipitating events, organ support, and liver transplantation. Despite intensive hospital care, the 28-day and 90-day mortality rates for patients with ACLF are 37.6% and 50.4%, respectively.^[6]^ However, ACLF remains a reversible syndrome, with the potential for recovery. The greatest opportunity to improve survival lies in early diagnosis and accurate prognosis, enabling optimal disease management.

Several established prognostic systems are currently used in patients with advanced liver disease and ACLF, including the Child–Turcotte–Pugh score,^[7]^ chronic liver failure-sequential organ failure assessment,^[8]^ and models for end-stage liver disease (MELD)^[9]^ and their derived scores.^[10,11]^ These tools have demonstrated important clinical value and are widely used for risk stratification. Nevertheless, their predictive performance may vary across patient populations, ACLF definitions, and clinical settings, leaving room for further improvement in individualized prognostic assessment. The present study was not designed to evaluate incremental predictive value over established prognostic scores, but rather to compare the performance and external stability of different modeling approaches developed within the same clinical dataset. Conventional scoring systems are generally based on predefined variables and may not fully capture complex relationships among demographic characteristics, clinical manifestations, complications, and laboratory indicators. Machine learning (ML), an important branch of artificial intelligence, can facilitate the analysis of complex relationships and interactions among multiple clinical variables. However, the performance of ML algorithms may still be influenced by class imbalance, feature scaling, data heterogeneity, sample size, and distributional differences between the development and validation cohorts.^[12,13]^ However, machine learning methods are not inherently superior to classical statistical models. Their performance may be influenced by sample size, class imbalance, feature scaling, data heterogeneity, overfitting, and distributional differences between development and validation cohorts. Therefore, rigorous external validation is essential when assessing the clinical utility of complex prediction models. Previous studies have explored the use of machine learning approaches for prognostic prediction in liver disease and ACLF.^[14-16]^ However, further evidence is needed regarding whether more complex algorithms provide stable and generalizable performance in patients with HBV-ACLF when compared with simpler and more interpretable models.

In the present study, we compared 6 predictive approaches for estimating 90-day mortality in patients with HBV-ACLF: logistic regression (LR), least absolute shrinkage and selection operator (LASSO)-based regression, support vector machine (SVM), decision tree (DT), random forest (RF), and K-nearest neighbor (KNN). LR was considered a classical statistical classification model, LASSO was considered a regularized regression-based approach, and SVM, DT, RF, and KNN were evaluated as machine learning algorithms. Using a 2-center retrospective cohort with an independent external validation set, we aimed to compare the discrimination, stability, and external generalizability of these approaches and to identify a practical model for clinical risk stratification in patients with HBV-ACLF.

## 2. Materials and methods

### 2.1. Study cohort and data collection

A total of 577 patients diagnosed with HBV-ACLF who were admitted to the First Affiliated Hospital of Nanchang University and Jiangxi Provincial People’s Hospital between January 2016 and October 2022 were retrospectively included. Of these, 513 patients from the First Affiliated Hospital of Nanchang University were randomly divided into a training group of 308 patients and a testing group of 205 patients, based on a 6:4 ratio. An additional 64 patients from Jiangxi Provincial People’s Hospital were used as an external validation group. Patients with HBV-ACLF were recruited based on the Asia–Pacific Association for the Study of the Liver criteria, which require hepatitis B surface antigen positivity for at least 6 months, a serum total bilirubin (TBIL) level of≥5 mg/dL, an international normalized ratio (INR) level ≥1.5, or prothrombin activity (PTA) of <40%, along with the presence of ascites and/or HE within 4 weeks. The inclusion criteria were as follows: age ≥16 years old; diagnosis of HBV-ACLF; and availability of clinical information. The exclusion criteria were as follows: HBV-ACLF coexisting with other chronic liver diseases; liver tumors or other malignancies; severe chronic extrahepatic diseases; previous liver transplantation; and human immunodeficiency virus infection or use of immunosuppressive drugs. This retrospective study was approved by the Ethics Committee of the First Affiliated Hospital of Nanchang University and Jiangxi Provincial People’s Hospital, and the need for informed consent was waived.

### 2.2. Data preparation, feature selection, and model training

The workflow for the development of ML models is illustrated in Figure 1. Thirty-three characteristics of patients with HBV-ACLF were retrospectively collected for analysis, including gender, age, weight, hypertension, diabetes, cirrhosis, portal hypertension, HE, hepatorenal syndrome (HS), glutamyltransferase, gastrointestinal bleeding (GIB), ascites, spontaneous bacterial peritonitis, electrolyte disorders, previous artificial liver support treatments, previous glucocorticoid therapy, and admission laboratory tests, namely, white blood cell (WBC) count, hemoglobin (HB) levels, platelet (PLT) count, alanine aminotransferase (ALT) levels, aspartate aminotransferase (AST) levels, TBIL levels, direct bilirubin levels, albumin (ALB) levels, serum creatinine (SCR) levels, glucose levels, triglyceride (TG) levels, total cholesterol (TC) levels, prothrombin time (PT), prothrombin activity (PTA), INR, fibrinogen levels, D-dimer levels, hepatitis B virus deoxyribonucleic acid (HBV-DNA). For variables with missing values, continuous variables were imputed using the median values calculated from the training cohort, whereas categorical variables were imputed using the most frequent category in the training cohort. The same imputation parameters were subsequently applied to the testing cohort and the external validation cohort.

**Figure 1.**
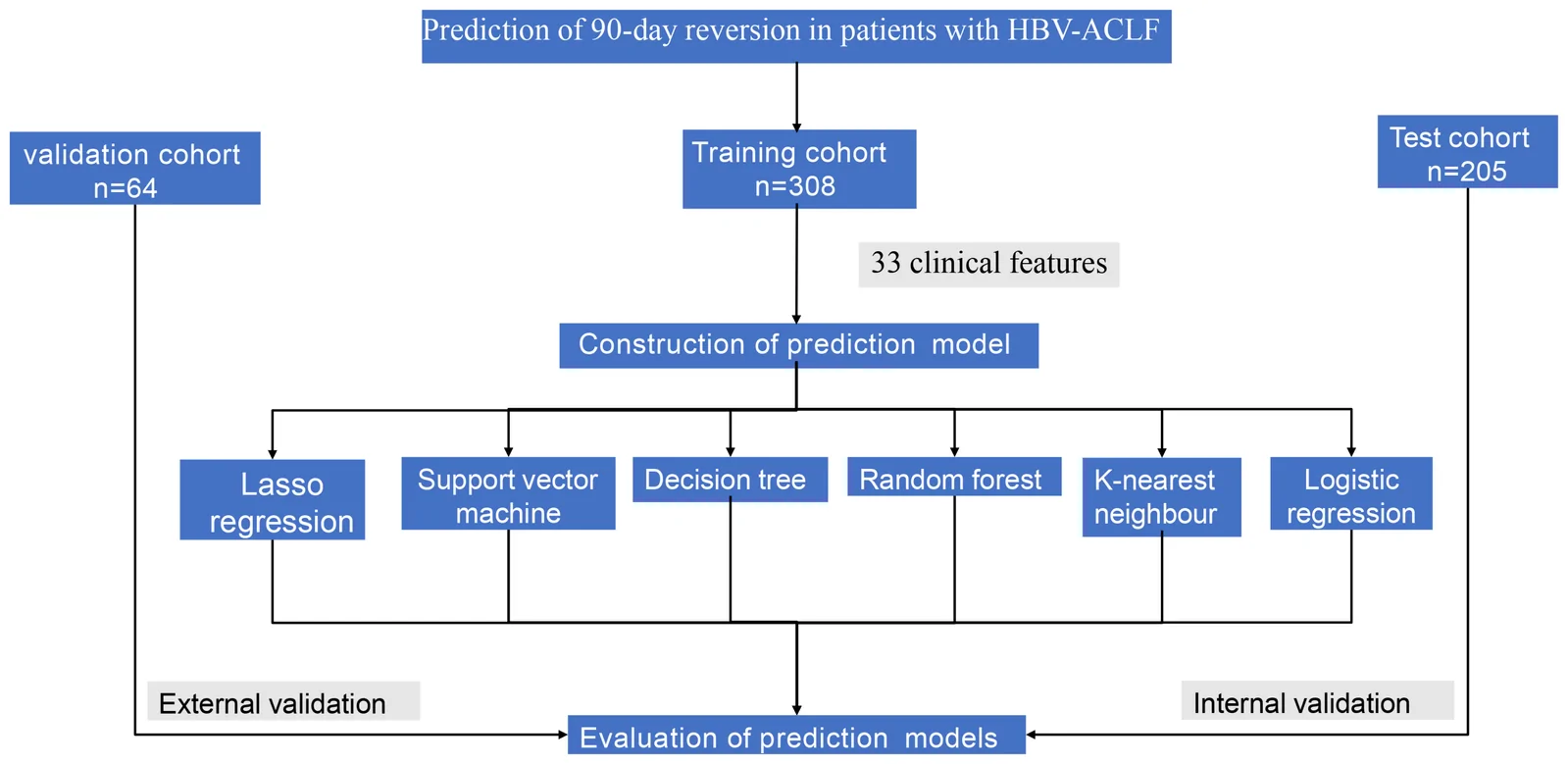
Flow of the study design. ACLF = acute-on-chronic liver failure, HBV = hepatitis B virus.

The prediction time point was defined as the index hospital admission. All candidate predictors were restricted to information available at this time, and no clinical information or treatment administered after the index admission was used for model development. The candidate predictors consisted of demographic characteristics, preexisting comorbidities, clinical manifestations, laboratory measurements obtained at admission, and previous treatment history. The latter included previous artificial liver support treatments and glucocorticoid therapy received before the index hospitalization, which represented historical information available at the prediction time point rather than interventions during the index hospitalization or subsequent follow-up.

ML is a statistical model in which a computer system performs tasks without using explicit instructions or reasoning.^[17]^ Generally, ML algorithms are divided into 2 categories, supervised and unsupervised learning. The supervised learning involves constructing a mathematical model from a dataset, known as training data, which contains inputs and desired outputs, referred to as supervisprimary peritonitised signals. In this study, selected features were applied to various supervised ML models, including LASSO, SVM, KNN, LR, DT and RF. In contrast, unsupervised learning uses a dataset containing only inputs, with the system recognizing patterns or structures in the data, such as grouping or clustering.

### 2.3. Support vector machine

SVM is a classical algorithm for classification, designed to address binary or multi-classification problems. Its core objective is to identify an optimal hyperplane in the feature space that maximizes the margin between classes. SVM performs well with small sample data, though its efficiency can be compromised when dealing with several variables. The “e1071” package in the R programming language was used to implement the SVM predictive model.

### 2.4. Decision tree and random forest

The use of the “rpart” package for decision analysis in the R programming language. DT is a classification and regression method named for its structure, where the rules and decisions resemble the trunk and branches of a tree. In DT, the predictor variable is represented as the root node, and the prediction result as the leaf node, with the path between them constituting the decision rule. The algorithm identifies the optimal variables and combinations to classify the data correctly. RF is an ensemble of DT that enhances the generalization ability, accuracy, and stability of the model while reducing overfitting, thereby improving predictive performance. For the RF model, the number of trees and split nodes were evaluated during model development. The final selected settings corresponded to 21 trees and 71 split nodes, which yielded the minimum reported error values in the training cohort.

### 2.5. K-nearest neighbor

The “knn” package in the R programming language was used to implement the KNN algorithm. The KNN algorithm measures the similarity between samples based on a distance metric. For a given sample to be classified, the algorithm calculates the distance between it and all neighboring training points, identifying the k loci to classify the variable as a close class. The *k* value and kernel function were evaluated during model development, and the final selected parameters were *k* = 11 and the “triangle” kernel.

### 2.6. Logistic regression

Logistic regression was performed in R using a conventional multivariable logistic regression model. Candidate predictors were evaluated using an AIC-based stepwise selection procedure, and the model with the lowest Akaike information criterion (AIC) was selected as the final logistic regression model. No L2 penalization was applied.

### 2.7. Least absolute shrinkage selection operator

The “glmnet” package in R was used to perform LASSO regression on the variables characterizing the cohort. In LASSO regression, the absolute value of the eigen coefficients gradually decreases as the lambda value increases, and eventually converges to 0. Initially, as the lambda value increases, the bias percentage decreases; however, beyond a certain point, it gradually increases. The optimal lambda value was selected within the training cohort using cross-validation. Predictors with non-zero coefficients at the selected lambda value were retained in the final LASSO model. The optimal lambda value corresponds to the minimum bias percentage.

### 2.8. Data preprocessing and prevention of data leakage

Before model development, the internal dataset was divided into the training and testing cohorts. Missing values were assessed before model development. For variables with missing data, continuous variables were imputed using the median values calculated from the training cohort, whereas categorical variables were imputed using the most frequent category in the training cohort. The imputation parameters derived from the training cohort were subsequently applied to the testing cohort and the external validation cohort. Binary categorical variables were encoded as 0 or 1. For nominal categorical variables with more than 2 categories, dummy variables were generated using a prespecified reference category. The same encoding scheme derived from the training cohort was applied consistently to the testing cohort and the external validation cohort.

For the LR, LASSO, SVM, and KNN models, continuous predictors were standardized using *Z*-score normalization. The mean and standard deviation of each variable were calculated exclusively from the training cohort, and the resulting transformation parameters were subsequently applied to the testing cohort and the external validation cohort. The DT and RF models were fitted using the original variable scales because tree-based splitting procedures are not dependent on the magnitude of the predictors. Before model development, the distributions and ranges of continuous variables were reviewed. Implausible values suggestive of data-entry errors were verified against the original medical records and corrected where necessary. Clinically plausible extreme values were retained because they may reflect disease severity and contain relevant prognostic information. No observations were excluded solely on the basis of statistical extremity. To prevent data leakage, imputation parameters, variable-scaling parameters, categorical-variable encoding rules, feature selection, and hyperparameter tuning were determined exclusively using the training cohort. The resulting preprocessing procedures and fitted models were then applied without modification to the testing cohort and the independent external validation cohort. Neither the testing cohort nor the external validation cohort was used for feature selection, model fitting, or hyperparameter optimization.

### 2.9. Statistical analysis

Data were analyzed using SPSS (version 21.0) and R (version 4.3.1). Categorical variables were expressed as frequencies and percentages and compared among the 3 cohorts using the chi-square test or Fisher exact test, as appropriate. Normally distributed continuous variables were expressed as the mean ± standard deviation and compared using 1-way analysis of variance (ANOVA). Non-normally distributed continuous variables were expressed as the median (interquartile range) and compared using the Kruskal–Wallis test. Model performance was assessed using accuracy, the area under the receiver operating characteristic curve (AUC), sensitivity, specificity, positive predictive value (PPV), and negative predictive value (NPV). The optimal classification threshold was determined from the ROC curve using the Youden index, defined as sensitivity + specificity − 1. The selected threshold was used to convert predicted probabilities into binary classifications for the calculation of accuracy, sensitivity, specificity, PPV, and NPV. A 2-sided *P* < .05 was considered statistically significant.

## 3. Results

### 3.1. Clinical characteristics

A total of 577 patients with HBV-ACLF were included in the study. Of these, 513 patients from the First Affiliated Hospital of Nanchang University were randomly assigned to the training group (n = 308) and the testing group (n = 205) in a 6:4 ratio. Additionally, 64 patients from Jiangxi Provincial People’s Hospital were used as the external validation group. In the training cohort, there were 270 males and 38 females, with an average age of 44.75 ± 12.26 years, and 130 deaths within 90 days. The testing group comprised 172 males and 33 females, with an average age of 44.38 ± 11.65 years, and 79 deaths within 90 days. The validation group had 41 males and 23 females, with an average age of 44.88 ± 13.29 years, and 31 deaths within 90 days. No statistically significant differences were observed in the measured baseline characteristics among the training, testing, and external validation cohorts (Table 1).

**Table 1:** HBV-ACLF patient characteristics in the training, test, and validation cohorts.

Characteristics	Training cohort (n = 308)	Test cohort (n = 205)	Validation cohort (n = 64)	*P* value
Gender				.377
Male	270	172	53	
Female	38	33	11	
Age (yr)	44.75 ± 12.26	44.38 ± 11.65	44.88 ± 13.29	.931
Weight (kg)	64.60 ± 10.55	64.91 ± 10.62	64.73 ± 11.23	.947
Hypertension				.603
No	269	184	58	
Yes	39	21	6	
Diabetes				.114
No	280	196	59	
Yes	28	9	5	
Cirrhosis				.425
No	202	133	47	
Yes	106	72	17	
Portal hypertension				.063
No	260	160	57	
Yes	48	45	7	
Hepatic encephalopathy				.270
No	214	154	49	
Yes	94	51	15	
Hepatorenal syndrome				.089
No	262	177	61	
Yes	46	28	3	
Gastrointestinal bleeding				.265
No	277	181	61	
Yes	31	24	3	
Ascites				.412
No	133	83	22	
Yes	175	122	42	
Spontaneous bacterial peritonitis				.120
No	122	85	27	
Yes	186	120	37	
Electrolyte disturbance				.903
No	176	113	36	
Yes	132	92	28	
Number of artificial liver treatments	1.00 (0, 2.00)	1.00 (0, 2.00)	0 (0, 2.00)	.305
Glucocorticoid therapy				.761
No	182	123	41	
Yes	126	82	23	
90-day survival				.355
Death	130	79	31	
Survival	178	126	33	
WBC (×10^9^/L)	6.76 ± 3.30	7.24 ± 3.67	6.54 ± 2.88	.190
HB (g/L)	129.50 ± 21.25	132.20 ± 19.34	129.14 ± 21.38	.303
PLT (×10^9^/L)	126.14 ± 56.85	125.74 ± 53.88	127.27 ± 57.06	.982
ALT (U/L)	721.59 ± 626.91	789.17 ± 714.56	606.66 ± 630.53	.141
AST (U/L)	522.32 ± 513.32	576.76 ± 577.35	480.03 ± 444.61	.346
TBIL (µmol/L)	293.70 (208.15, 402.03)	299.60 (208.85, 377.00)	262.35 (158.23, 345.25)	.115
DBIL (µmol/L)	189.05 ± 91.84	181.20 ± 81.88	161.09 ± 103.24	.072
ALB (g/L)	32.31 ± 4.14	32.40 ± 4.38	31.03 ± 4.45	.190
SCR (µmol/L)	74.50 ± 32.21	75.13 ± 40.73	78.66 ± 44.79	.715
Glu (mmol/L)	5.53 ± 4.05	4.65 ± 2.25	5.10 ± 3.34	.733
TG (mmol/L)	1.10 ± 0.72	1.14 ± 0.67	1.18 ± 1.28	.722
TC (mmol/L)	2.51 ± 0.91	2.67 ± 0.95	2.63 ± 1.32	.174
PT (s)	23.30 (19.40, 30.70)	24.50 (19.75, 29.85)	24.90 (21.78, 29.13)	.694
PTA (%)	31.75 (23.70, 40.20)	31.30 (24.00, 39.40)	36.00 (30.00, 38.00)	.346
INR	2.13 (1.76, 2.79)	2.17 (1.73, 2.71)	2.16 (2.05, 2.51)	.289
FIB (g/L)	1.33 ± 1.11	1.42 ± 1.57	1.85 ± 2.68	.050
d-Dimer (mg/L)	2.48 ± 2.60	3.00 ± 5.48	2.16 ± 2.26	.194
HBV-DNA (log10 IU/mL)	5.18 (3.44, 6.94)	5.58 (3.73, 6.85)	5.66 (1.00, 7.14)	.613

ALB = albumin, ALT = alanine aminotransferase, AST = aspartate aminotransferase, DBIL = direct bilirubin, FIB = fibrinogen, Glu = glucose, HB = hemoglobin, HBV-DNA = hepatitis B virus DNA, INR = international normalized ratio, PLT = platelets, PT = prothrombin time, PTA = prothrombin activity, SCR = serum creatinine, TBIL = total bilirubin, TC = total cholesterol, TG = triglyceride, WBC = white blood cell.

### 3.2. LR-based predictive model

It was observed that the regression model achieved the best fit, with an Akaike Information Criterion value of 250, when the predictive model included the following variables: glutamyltransferase, SCR, diabetes, fibrinogen, TBIL, ALT, AST, HE, systolic blood pressure (SBP), HS, GIB, and INR as determined through LR analysis (Fig. 2A). The complete specification of the final logistic regression model, including the intercept and regression coefficients, is provided in Table S1, Supplemental Digital Content 1. In the training cohort, the regression prediction model had an AUC value of 0.905, a sensitivity of 0.754, and a specificity of 0.902. Positive predictive value (PPV) and negative predictive value (NPV) were 0.841 and 0.842, respectively (Fig. 2B). In the testing cohort, the predictive model had an AUC of 0.928, with sensitivity and specificity values of 0.867 and 0.884, respectively, and PPV and NPV of 0.837 and 0.906, respectively (Fig. 2C). For the validation cohort, the predictive model had an AUC of 0.849, with sensitivity and specificity values of 0.645 and 0.909, respectively. PPV and NPV in the validation cohort were 0.870 and 0.732, respectively (Fig. 2D).

**Figure 2.**
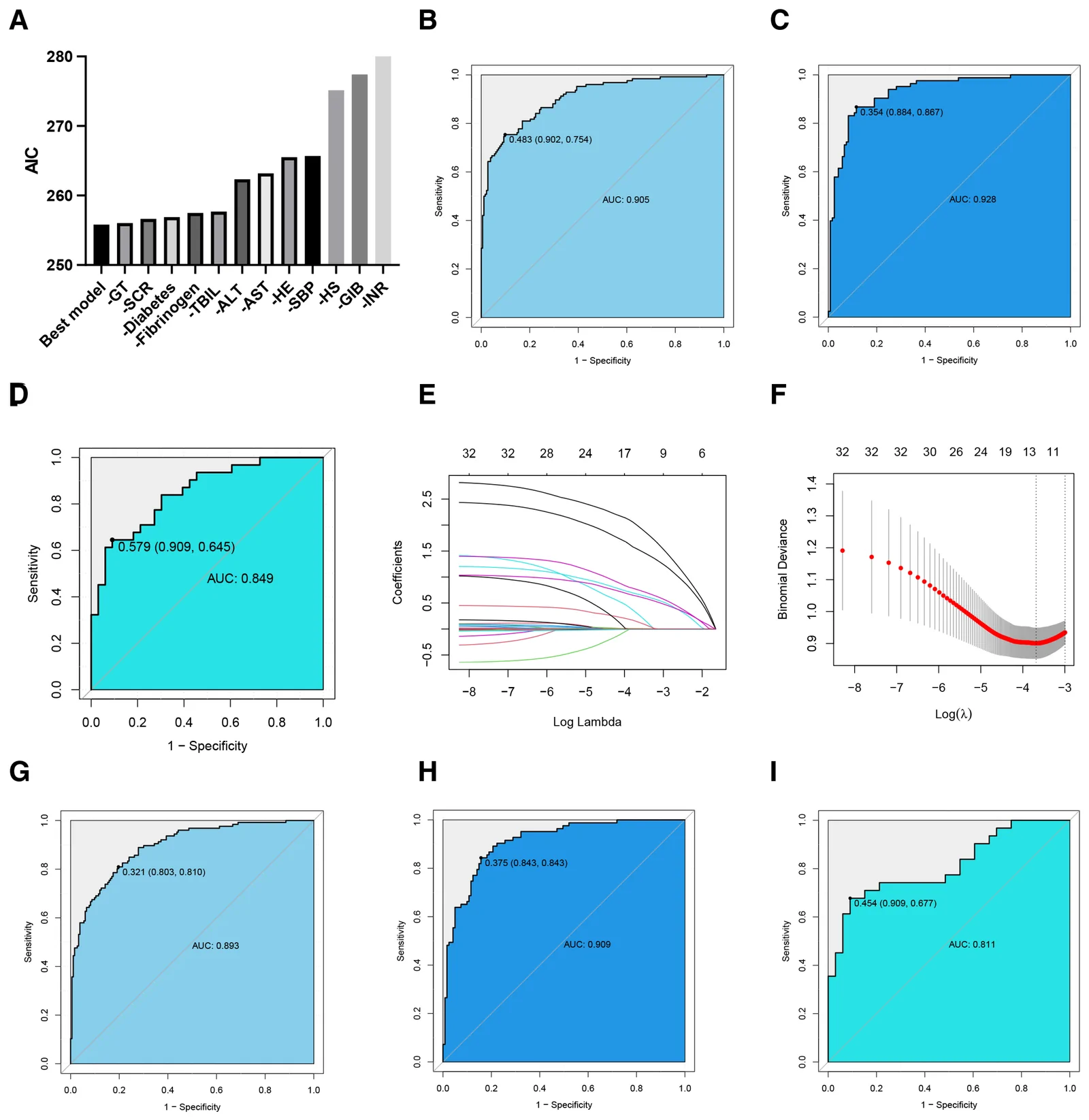
LR and LASSO-based capability assessment of the predictive models. (A) Best predictive model and each feature’s AIC value; (B) the ROC curve of the LR predictive model in the training cohort; (C) the ROC curve of the LR predictive model in the testing cohort; (D) the ROC curve of the LR predictive model in the validation cohort. (E) Relationship between the lambda values and clinical feature coefficients; (F) relationship between the lambda values and bias percentage of clinical features. (G) The ROC curve of the LASSO predictive model in the training cohort; (H) the ROC curve of the LASSO predictive model in the testing cohort; (I) the ROC curve of the LASSO predictive model in the validation cohort. AIC = Akaike information criterion, ALT = alanine aminotransferase, AST = aspartate aminotransferase, AUC = area under the curve, GIB = gastrointestinal bleeding, GT = glutamyltransferase, HE = hepatic encephalopathy, HS = hepatorenal syndrome, INR = international normalized ratio, ROC = receiver operating characteristic, SCR = serum creatinine, TBIL = total bilirubin.

### 3.3. LASSO-based predictive model

The clinical characteristics of the training cohort were analyzed using LASSO regression. As the lambda value increased, the absolute values of the characteristic coefficients gradually decreased, eventually converging to 0. The bias percentage followed a similar trend, first decreasing and then gradually increasing with higher lambda values. The key indicators identified through this process were GIB, age, SBP, HE, HS, HB, TBIL, PTA, and INR. A visual representation of the indicator screening process is provided (Fig. 2E, F). In the training cohort, the predictive model developed using LASSO regression analysis yielded an AUC value of 0.893, with a sensitivity of 0.810 specificity of 0.803, PPV of 0.739, and NPV of 0.860 (Fig. 2G). In the testing cohort, the predictive model demonstrated an AUC value of 0.909, with a sensitivity and specificity of 0.843 each, and PPV and NPV of 0.787 and 0.887, respectively (Fig. 2H). For the validation cohort, the model achieved an AUC value of 0.811, with a sensitivity of 0.677, specificity of 0.909, PPV of 0.875, and NPV of 0.750 (Fig. 2I).

### 3.4. SVM‑based predictive model

In the 90-day survival prediction model for HBV-ACLF constructed using SVM, it was observed that the model achieved the highest accuracy when the number of vectors was 6 (Fig. 3A). These 6 indicators included GIB, HE, HS, INR, PTA, and PT. In the training cohort, the predictive model had an AUC value of 0.881, with a sensitivity of 0.794, specificity of 0.842, PPV of 0.775, and NPV of 0.856 (Fig. 3C). In the testing cohort, the predictive model had an AUC of 0.865, sensitivity of 0.783, specificity of 0.843, PPV of 0.774, and NPV of 0.850, respectively (Fig. 3D). For the validation cohort, the predictive model achieved an AUC of 0.762, with a sensitivity of 0.581, specificity of 0.909, PPV of 0.857, and NPV of 0.700 (Fig. 3E).

**Figure 3.**
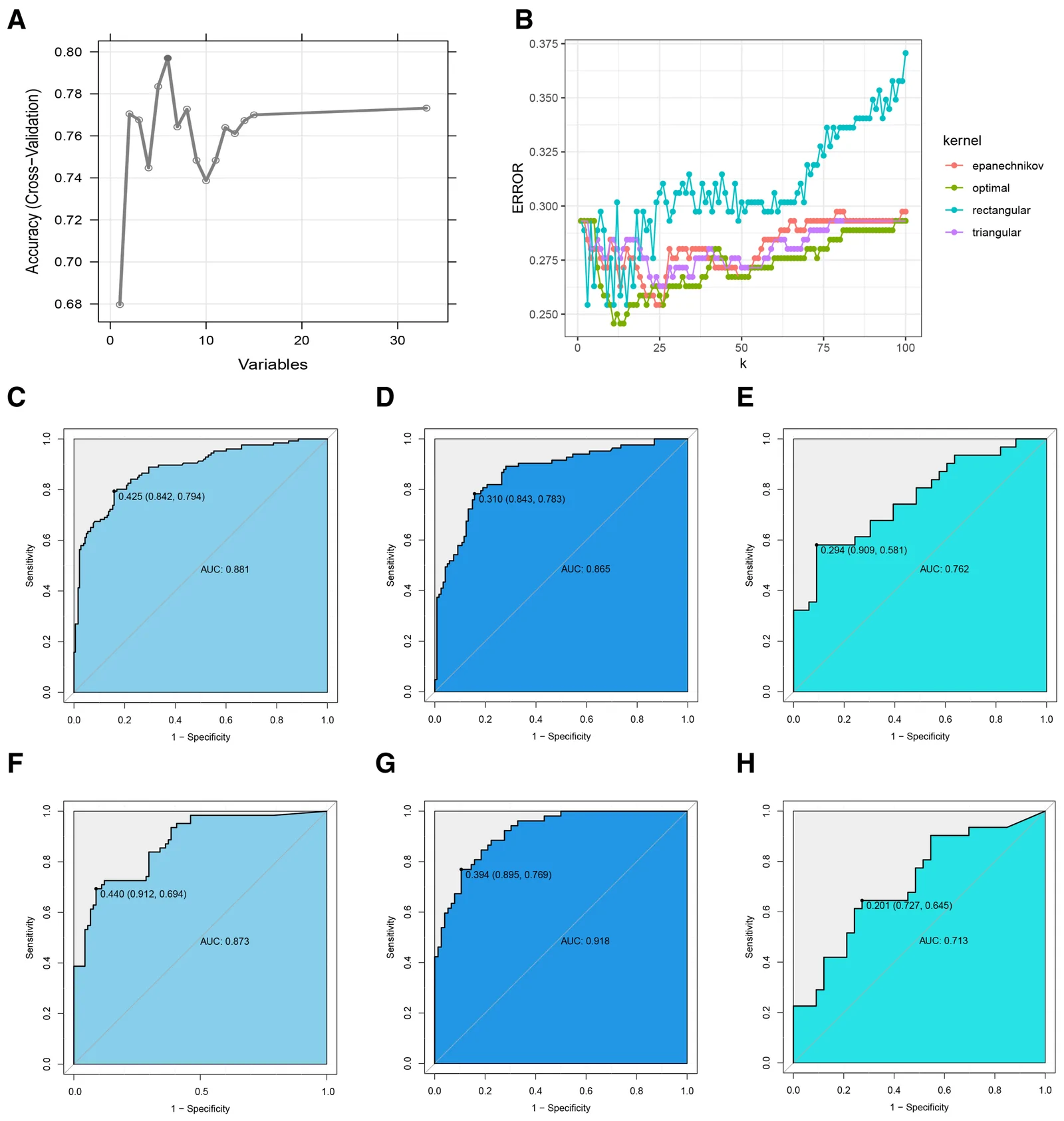
SVM and KNN-based capability assessment of the predictive models. (A) Number of vectors and model accuracy of the SVM. (B) Screening for the best kernel function and *k*-value. (C) The ROC curve of the SCM predictive model in the training cohort; (D) the ROC curve of the SVM predictive model in the testing cohort; (E) the ROC curve of the SVM predictive model in the validation cohort. (F) The ROC curve of the KNN predictive model in the training cohort; (G) the ROC curve of the KNN predictive model in the testing cohort; (H) the ROC curve of the KNN predictive model in the validation cohort. AUC = area under the curve, KNN = K nearest neighbor, ROC = receiver operating characteristic, SVM = support vector machine.

### 3.5. KNN‑based predictive model

It was observed that the optimal kernel function for the prediction model was ‘triangle’, and the optimal k-value was 11 after optimizing the hyperparameters of the KNN function (Fig. 3B). The prediction model was constructed based on these conditions. In the training cohort, the predictive model achieved an AUC value of 0.873, with a sensitivity of 0.694, specificity of 0.912, PPV of 0.843, and NPV of 0.814 (Fig. 3F). In the testing cohort, the predictive model yielded an AUC value of 0.918, with a sensitivity of 0.769, specificity of 0.895, PPV of 0.831, and NPV of 0.850 (Fig. 3G). For the validation cohort, the predictive model had an AUC value of 0.713, with a sensitivity of 0.645, specificity of 0.727, PPV of 0.690, and NPV of 0.686 (Fig. 3H).

### 3.6. DT-based predictive model

In the DT prognostic model, 7 best splitting nodes were identified (Fig. 4A) and the variable features of the constructed predictive model were ranked according to their importance. The top 5 features were GIB, HS, PTA, HE, and HBV-DNA (Fig. 4B). These top 5 important features were selected to construct the DT prediction model. In the training cohort, the predictive model achieved an AUC value of 0.853, with a sensitivity of 0.770, specificity of 0.885, PPV of 0.822, and NPV of 0.848 (Fig. 4C). In the testing cohort, the predictive model recorded an AUC value of 0.828, with a sensitivity of 0.783, specificity of 0.835, PPV of 0.765, and NPV of 0.849 (Fig. 4D). For the validation cohort, the predictive model had an AUC value of 0.760, with a sensitivity of 0.581, specificity of 0.909, PPV of 0.857, and NPV of 0.700 (Fig. 4E).

**Figure 4.**
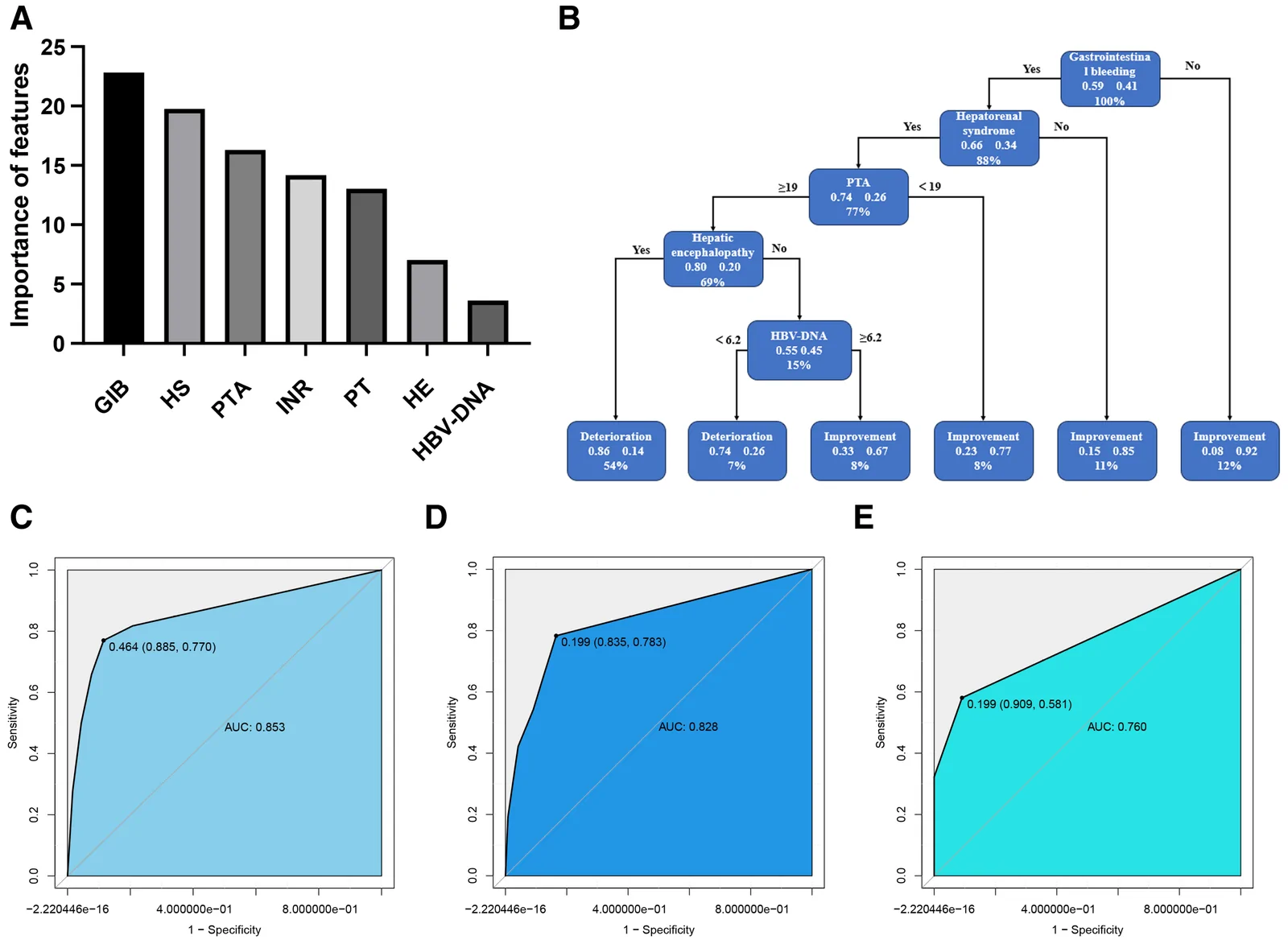
DT-based capability assessment of the predictive model. (A) The significance of clinical features and radiomic features for DT-based predictive model; (B) DT diagram; (C) the ROC curve of the DT predictive model in the training cohort; (D) the ROC curve of the DT predictive model in the testing cohort; (E) the ROC curve of the DT predictive model in the validation cohort. DT = decision tree, GIB = gastrointestinal bleeding, HBV-DNA = hepatitis B virus DNA, HE = hepatic encephalopathy, HS = hepatorenal syndrome, INR = international normalized ratio, PT = prothrombin time, PTA = prothrombin activity.

### 3.7. RF-based predictive model

Through the RF prediction model, it was observed that the out-of-bag error reached its minimum value of 0.23 when the model incorporated 21 trees (Fig. 6A). Further analysis revealed that the error decreased to a minimum value of 0.186 when the number of split nodes was 71 (Fig. 6B). Additionally, the feature importance among the 33 features in the model was analyzed. It was found that GIB, HS, and INR significantly influenced prediction accuracy. Furthermore, GIB, HS, and HE played a significant role in reducing the Gini coefficient of the prediction model (Fig. 5A, B). In the training cohort, the predictive model demonstrated an AUC value of 1, with sensitivity, specificity, PPV, and NPV, each being 1 (Fig. 6C). In the testing cohort, the model had an AUC value of 0.912, with a sensitivity of 0.952, specificity of 0.802, PPV of 0.767, and NPV of 0.960 (Fig. 6D). In the validation cohort, the model achieved an AUC value of 0.728, with a sensitivity of 0.839, specificity of 0.545, PPV of 0.605, and NPV of 0.783 (Fig. 6E).

**Figure 5.**
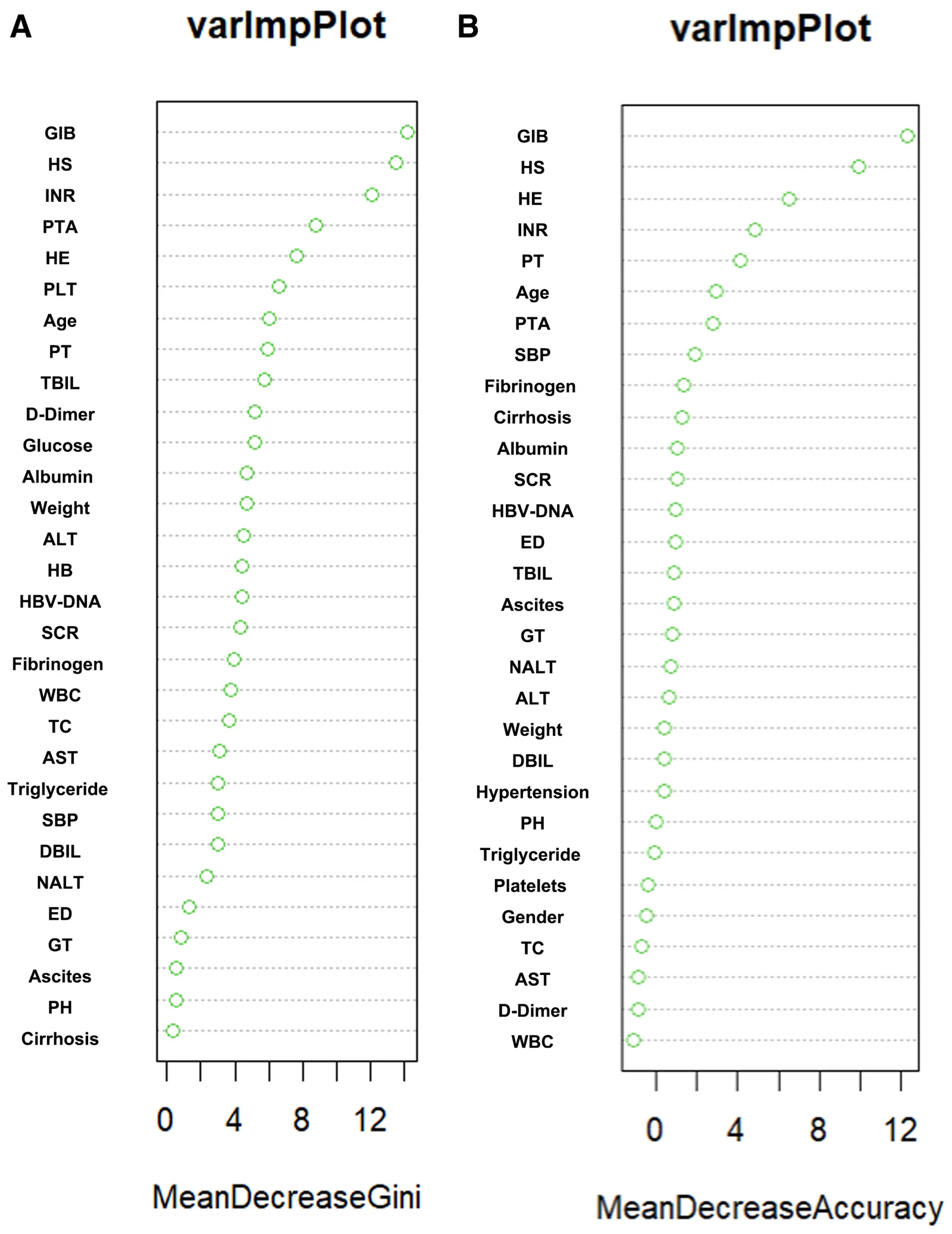
Importance of radiomic and clinical features in reducing the Gini coefficien (A) and improving the model accuracy (B). ALT = alanine aminotransferase, AST = aspartate aminotransferase, DBIL = direct bilirubin, ED = electrolyte disorders, GIB = gastrointestinal bleeding, GT = glutamyltransferase, HB = hemoglobin, HBV-DNA = hepatitis B virus DNA, HE = hepatic encephalopathy, HS = hepatorenal syndrome, INR = international normalized ratio, PH = portal hypertension, PLT = platelets, PT = prothrombin time, PTA = prothrombin activity, RF = random forest, SCR = serum creatinine, TBIL = total bilirubin, TC = total cholesterol, WBC = white blood cells.

**Figure 6.**
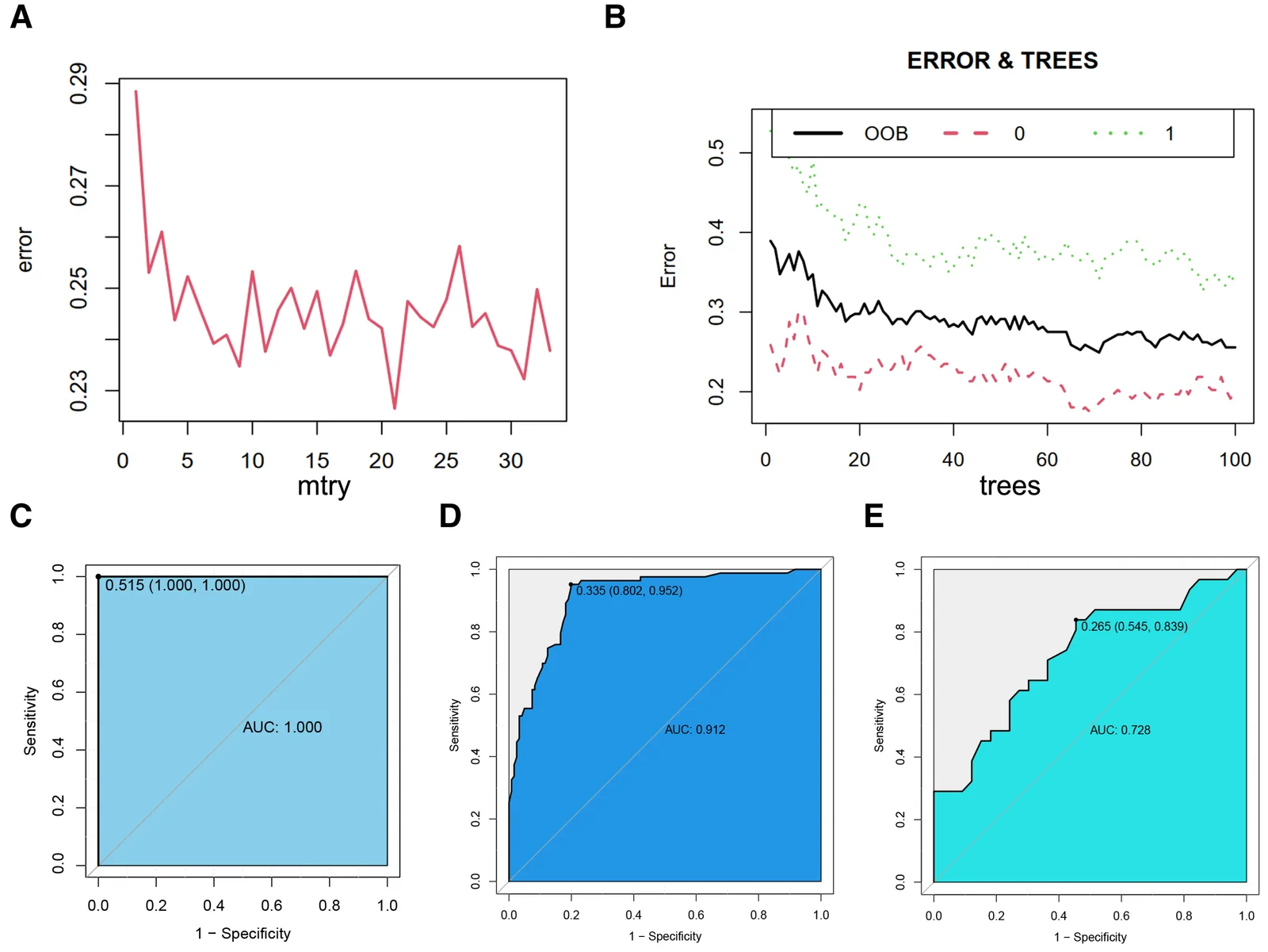
RF-based capability assessment of the predictive model. (A) Relationship between the number of split points and error in the tree. (B) Relationship between the number of trees and OOB error in RF. (C) The ROC curve of the RF predictive model in the training cohort. (D) The ROC curve of the RF predictive model in the testing cohort; (E) the ROC curve of the RF in the validation cohort. AUC = area under the curve, RF = random forest, ROC = receiver operating characteristic.

### 3.8. Consensus predictors across different ML models

Although the 6 predictive approaches employ different modeling principles, they exhibited considerable consistency in feature selection. Specifically, 5 clinical variables were repeatedly identified as top predictors across 3 or more models: INR, HE, GIB, PT/PTA, and BIL. INR was selected by LR, LASSO, SVM, and RF. HE was included in LR, LASSO, DT, and RF. GIB was selected by LR, LASSO, SVM, DT, and RF – making it the most universally selected predictor. PT/PTA appeared in LR, SVM, and RF, while TBIL was chosen by LR, LASSO, and KNN. The convergence of these 5 variables across diverse algorithmic architectures strongly suggests that they represent truly robust, model-independent prognostic signals rather than algorithm-specific artifacts.

### 3.9. Comparison of the predictive abilities of various models

The predictive ability of the HBV-ACLF prognostic models constructed using multiple ML methods is presented in Table 2. In the training cohort, the RF prediction model outperformed the LASSO, LR, SVM, DT, and KNN prediction models, showing significantly higher AUC, sensitivity, specificity, PPV, and NPV. In the training cohort, the RF model showed the highest apparent predictive performance, with an AUC, sensitivity, specificity, PPV, and NPV of 1.000. Its performance remained relatively good in the internal testing cohort, with an AUC of 0.912, sensitivity of 0.952, specificity of 0.802, PPV of 0.767, and NPV of 0.960. However, a marked decline was observed in the independent external validation cohort, in which the AUC, sensitivity, specificity, PPV, and NPV were 0.728, 0.839, 0.545, 0.605, and 0.783, respectively. This substantial decrease from the training and testing cohorts to the external validation cohort suggested limited external generalizability and possible overfitting of the RF model. In the training cohort, the LASSO prediction model exhibited the lowest PPV (0.739), while the KNN prediction model had the lowest sensitivity (0.694). In the testing and validation cohorts, the SVM and DT prediction models showed the lowest sensitivity, both recording a value of 0.581. Although the LR model did not achieve the perfect apparent performance observed for the RF model in the training cohort, it demonstrated more consistent discrimination across the training, testing, and external validation cohorts. In particular, the LR model outperformed the RF model in the external validation cohort, with an AUC of 0.849 versus 0.728, the LR model had a numerically higher AUC than the RF model in the external validation cohort (0.849 vs 0.728). Given the limited size of the external validation cohort, this numerical difference should be interpreted cautiously and was not considered evidence of statistical superiority. Notably, the LR prediction model exhibited more stable prediction performance across all cohorts, making it a reliable choice among the models evaluated.

**Table 2:** Comparison of the capabilities of ML prediction models for 90-day reversion in HBV-ACLF patients.

Characteristics	LR	LASSO	SVM	KNN	DT	RF
Training cohort						
Accuracy	0.841	0.806	0.822	0.824	0.838	1.000
AUC	0.905	0.893	0.881	0.873	0.853	1.000
Sensitivity	0.754	0.810	0.794	0.694	0.770	1.000
Specificity	0.902	0.803	0.842	0.912	0.885	1.000
PPV	0.841	0.739	0.775	0.843	0.822	1.000
NPV	0.842	0.860	0.856	0.814	0.848	1.000
Test cohort						
Accuracy	0.877	0.843	0.819	0.843	0.814	0.863
AUC	0.928	0.909	0.865	0.918	0.828	0.912
Sensitivity	0.867	0.843	0.783	0.769	0.783	0.952
Specificity	0.884	0.843	0.843	0.895	0.835	0.802
PPV	0.837	0.787	0.774	0.831	0.765	0.767
NPV	0.906	0.887	0.850	0.850	0.849	0.960
Validation cohort						
Accuracy	0.781	0.797	0.750	0.688	0.750	0.688
AUC	0.849	0.811	0.762	0.713	0.760	0.728
Sensitivity	0.645	0.677	0.581	0.645	0.581	0.839
Specificity	0.909	0.909	0.909	0.727	0.909	0.545
PPV	0.870	0.875	0.857	0.690	0.857	0.605
NPV	0.732	0.750	0.700	0.686	0.700	0.783

AUC = area under the curve, DT = decision tree, KNN = K nearest neighbor, LASSO = least absolute shrinkage selection operator, LR = logistic regression, NPV = negative predictive value, PPV = positive predictive value, RF = random forest, SVM = support vector machine.

## 4. Discussion

In this study, we aimed to compare multiple predictive approaches, including classical logistic regression and several machine learning algorithms, for estimating 90-day mortality in patients with HBV-ACLF. Our results demonstrate that logistic regression, despite its relative simplicity, provided the most stable and consistent predictive performance across both the internal testing cohort and the independent external validation cohort. More complex machine learning models, including random forest, support vector machine, decision tree, and K-nearest neighbor, showed variable performance and in some cases experienced substantial declines in predictive accuracy when applied to external data.

Previous studies have demonstrated that conventional statistical modeling techniques are widely used and increasingly accepted for prognostic prediction in ACLF.^[18,19]^ However, these traditional methods have several limitations. First, their ability to process complex features is restricted.^[20]^ Variable selection in conventional models often depends on the experience and prior knowledge of the researcher. For example, in predicting ACLF mortality, researchers might rely on commonly known liver function indicators and demographic predictors, potentially overlooking important underlying variables or including those with minimal impact on mortality. This subjectivity can reduce the predictive power of the model. Furthermore, when handling large, high-dimensional datasets – such as clinical, genetic, and imaging data – traditional statistical models struggle to efficiently filter out the most relevant predictive features.^[21]^ Secondly, conventional statistical models lack the flexibility and generalisability required to adapt to diverse datasets.^[20,22]^ Different patient populations might exhibit varying data patterns, and the distribution of new data might differ from that used in model training. As a result, traditional models often perform poorly when applied to new or varied data, limiting their predictive accuracy.^[23]^ ML predictive models use data-driven techniques and have been shown to either match or surpass the performance of traditional statistical methods. For instance, Zheng et al^[24]^ and Hou et al^[25]^ developed and validated artificial neural network-based models for predicting mortality in patients with HBV-ACLF, while Shi et al^[26]^ developed and validated a categorical regression tree model that outperformed the MELD score in short-term mortality prediction. In this study, it was found that ML prediction models similarly demonstrated strong predictive performance, with the LR prediction model showing the most stable performance.

Prediction models were developed using 6 ML tools: LR, SVM, DT, RF, KNN, and LASSO. Our results indicate that the RF prediction model performed better overall compared to LR, SVM, DT, KNN, and LASSO in the training cohort. However, although the RF model maintained relatively good discrimination in the internal testing cohort, with an AUC of 0.912, its performance declined substantially in the independent external validation cohort. In the external validation cohort, the AUC decreased to 0.728, while specificity and PPV decreased to 0.545 and 0.605, respectively. This suggests the RF model is prone to predicting a higher number of false positives, potentially due to the uneven distribution of dichotomous variables. A previous study on end-stage liver disease also demonstrated that the RF prediction model significantly outperformed the SVM and DT prediction models in terms of performance metrics.^[23]^ Interestingly, while the LR model was less effective than RF in the training cohort, it outperformed RF in the testing and validation cohorts. The RF model achieved perfect discrimination in the training cohort but exhibited a marked decline in performance in both the testing and external validation cohorts. The marked decline in external validation performance suggests that the RF model had limited generalizability despite its excellent apparent performance in the training cohort.. This decline is likely attributable to methodological characteristics of the RF algorithm rather than differences in variable distribution alone. Although RF is capable of capturing complex nonlinear relationships and interactions among predictors, its high flexibility may increase the risk of fitting cohort-specific patterns, particularly in datasets of moderate size. As an ensemble learning method, RF may also exhibit relatively high variance, leading to reduced stability when applied to independent populations. These characteristics likely contributed to the discrepancy between the excellent training performance and the substantially lower validation performance observed in the present study. The findings underscore the importance of external validation and suggest that increased model complexity does not necessarily result in superior generalizability. This discrepancy might be attributed to the small sample size or substantial fluctuations in the data distribution within these cohorts. Additionally, the random sampling and feature selection in RF could result in substantial variability between decision trees, affecting the stability of the model.

An important finding of the present study is that LR demonstrated the most stable performance across the testing and external validation cohorts despite its relative simplicity compared with several machine learning algorithms. Although complex machine learning approaches are capable of modeling nonlinear relationships and high-order interactions, greater model complexity does not necessarily result in superior predictive performance. In datasets of moderate size, more complex models may be more susceptible to overfitting and may capture cohort-specific patterns that do not generalize well to independent populations. In contrast, LR provides a parsimonious and highly interpretable framework that may improve robustness and generalizability. The relatively consistent discrimination observed for the LR model across the 3 cohorts suggests that simpler models may remain valuable for clinical prediction. However, given the limited external validation sample size, these findings should not be interpreted as demonstrating statistical superiority of LR over the other models.

Our study also found that not all ML prediction models are good predictors of prognosis for HBV-ACLF. Although the DT prediction model demonstrated relatively good accuracy across the training, testing, and validation cohorts, its AUC was lower compared to the 5 other models in the training and testing cohorts. One limitation of the DT model is its simplicity as a nonparametric classifier, which does account for interactions between variables during tree formation since only a single variable is used at each screening.^[27]^ Similarly, the performance metrics of the SVM prediction model were comparable to the DT prediction model. The KNN prediction model exhibited good accuracy in the training and testing cohorts; however, its PPV and NPV in the validation cohort were 0.690 and 0.689, respectively, suggesting a relatively high error rate when predicting mortality in patients with HBV-ACLF. The LASSO predictive model, while demonstrating better predictive performance than some other models, still underperformed compared to the LR model. The poorer performance of the DT, SVM, KNN, and LASSO models could be attributed to their inherent challenges, such as susceptibility to overfitting, high computational complexity, and sensitivity to feature scaling.

A recent study demonstrated the use of ML in predicting prognosis after liver transplantation in patients with ACLF, emphasizing its potential in this field.^[15]^ Another study reported that ML could significantly aid in predicting cardiovascular mortality in patients with nonalcoholic steatohepatitis after transplantation.^[28]^ With the development of technology, the application of ML is expanding beyond prognostic prediction. Chang et al^[29]^ reported that ML models outperformed traditional scoring systems in identifying clinically significant stages of nonalcoholic fatty liver disease-related liver fibrosis and cirrhosis. Moreover, ML has been shown to predict venous thrombosis in patients with cirrhosis.^[30]^ Additionally, Zhou et al^[31]^ demonstrated that ML can be used to model the grading of chronic hepatitis B inflammation, a key factor in clinical diagnosis and treatment.

The relatively poorer performance of several alternative approaches may also be explained by their methodological characteristics. Although SVM can effectively model complex decision boundaries, its performance is highly dependent on kernel selection and parameter tuning and may be sensitive to differences in data distribution between cohorts. KNN is a distance-based algorithm and may be adversely affected by high-dimensional feature spaces, variable noise, and heterogeneity among patients, which can reduce its generalizability. DT models are highly interpretable but are prone to instability and overfitting, particularly in datasets of moderate size, as small changes in the training data may lead to substantial changes in tree structure. LASSO regression can reduce model complexity through coefficient shrinkage and variable selection; however, the regularization process may exclude predictors that contain additional prognostic information, potentially resulting in lower predictive performance compared with conventional logistic regression. These findings suggest that the methodological characteristics of individual algorithms should be carefully considered when developing prognostic models for HBV-ACLF.

Although this is a retrospective study, we used readily available features, investigated multiple ML methods to select the best ML model, and validated the model in an independent external validation set. Although no statistically significant differences in baseline characteristics were observed among the 3 cohorts, the independent external validation cohort was derived from a different institution, providing an additional assessment of the model’s generalizability. These findings suggest that the proposed prediction model has the potential to assist prognostic risk stratification in patients with HBV-ACLF. However, further multicentre prospective studies with larger external validation cohorts and comprehensive model evaluation are warranted before routine clinical application. Of course, this study has certain limitations. First, this was a retrospective study, which may be subject to selection bias and unmeasured confounding. Second, although an independent external validation cohort was included, the sample size of the external validation cohort was relatively modest, which may limit the stability and generalizability of the validation results. Third, the more complex machine learning models, particularly random forest, demonstrated a decline in performance during external validation, suggesting a potential risk of overfitting despite their strong performance in the training cohort. Forth, cross-validation was not used during model tuning in the original analysis. Although parameter selection was restricted to the training cohort, future studies should incorporate resampling-based procedures such as k-fold cross-validation to provide a more robust assessment of model stability and reduce the risk of overfitting. Fifth, the present study focused primarily on discrimination and comparative model performance. Calibration, formal statistical comparisons between ROC curves, and decision-curve analysis were not performed. Therefore, the observed numerical differences between models should not be interpreted as evidence of statistical superiority or clinical utility. In particular, the relatively small external validation cohort may limit the precision and stability of model-performance estimates. These aspects require further evaluation in larger independent multicenter cohorts before clinical implementation. Future studies may incorporate methods such as the DeLong test to further evaluate differences in predictive performance among competing models. In addition, the predictive models were not directly compared with established prognostic scoring systems, such as MELD, MELD-Na, or ACLF-specific scores. Therefore, the present study cannot determine whether any of the evaluated models provides incremental predictive value beyond currently available clinical tools. This limits conclusions regarding their clinical usefulness and should be addressed in future prospective studies with complete data for established prognostic scores. Future multicenter prospective studies with larger validation cohorts, comprehensive calibration assessment, formal model comparisons, decision-curve analysis, resampling-based validation strategies, and dynamic longitudinal data are warranted to further evaluate the robustness and clinical applicability of prognostic models for HBV-ACLF.

## 5. Conclusion

In summary, LR, a simpler and more interpretable model, showed relatively consistent discrimination across the internal testing and external validation cohorts. However, given the limited size of the external validation cohort, these findings should be interpreted cautiously and do not establish statistical superiority of LR over the other evaluated approaches. Further validation in larger, multicenter cohorts is warranted.

## Author contributions

**Conceptualization:** Yin Zhu, An Liang.

**Data curation:** Juan Liu, Wentao Zhu, Ting Deng, Yin Zhu, Qinglang Xu, Pei Shi, Jiwei Fu.

**Formal analysis:** Juan Liu, Yin Zhu, Qinglang Xu, Pei Shi.

**Funding acquisition:** Xiaoping Wu.

**Investigation:** Wentao Zhu, Qinglang Xu, An Liang.

**Methodology:** Wentao Zhu, Ting Deng, Yin Zhu.

**Project administration:** Ting Deng, Yuna Wang, Xiaoping Wu.

**Resources:** Ting Deng, Qinglang Xu, An Liang, Yuna Wang.

**Software:** Yin Zhu, Pei Shi, An Liang, Xiaoping Wu.

**Supervision:** Wentao Zhu, An Liang, Jiwei Fu, Xiaoping Wu.

**Validation:** Pei Shi, Yuna Wang, Jiwei Fu.

**Visualization:** Yuna Wang.

**Writing – original draft:** Juan Liu.

**Writing – review & editing:** Jiwei Fu, Xiaoping Wu.


