# Supplementary material for: Predicting 90-day mortality in patients with HBV-ACLF: A comparison of machine learning models and logistic regression
Source: Medicine (Baltimore). 2026 Sep 25;105(39):e50789. doi: 10.1097/MD.0000000000050789 (PMC13619297; doi:10.1097/MD.0000000000050789)
Supplement: Supplementary file 1 [file medi-105-e50789-s001.docx]

Supplementary Table S1. Final logistic regression model specification

| **Predictor** | **Regression coefficient (β)** |
| --- | --- |
| Intercept | −6.314945 |
| Gastrointestinal bleeding | 2.705251 |
| Diabetes | 1.151971 |
| Spontaneous bacterial peritonitis | 1.348340 |
| Hepatic encephalopathy | 1.467675 |
| Hepatorenal syndrome | 2.446796 |
| Previous glucocorticoid therapy | −0.582496 |
| TBIL | 0.002865 |
| ALT | −0.001474 |
| AST | 0.001817 |
| Serum creatinine | −0.011404 |
| INR | 1.322431 |
| Fibrinogen | 0.761139 |

The predicted probability of 90-day mortality was calculated as P=1/[1+exp(−LP)], where LP represents the linear predictor constructed from the intercept and regression coefficients shown above.

| **Predictor** | **Regression coefficient (β)** |
| --- | --- |
| Intercept | −6.314945 |
| Gastrointestinal bleeding | 2.705251 |
| Diabetes | 1.151971 |
| Spontaneous bacterial peritonitis | 1.348340 |
| Hepatic encephalopathy | 1.467675 |
| Hepatorenal syndrome | 2.446796 |
| Previous glucocorticoid therapy | −0.582496 |
| TBIL | 0.002865 |
| ALT | −0.001474 |
| AST | 0.001817 |
| Serum creatinine | −0.011404 |
| INR | 1.322431 |
| Fibrinogen | 0.761139 |
